# Cross-sectional study of availability and pharmaceutical quality of antibiotics requested with or without prescription (Over The Counter) in Surabaya, Indonesia

**DOI:** 10.1186/1471-2334-10-203

**Published:** 2010-07-09

**Authors:** Usman Hadi, Peterhans van den Broek, Erni P Kolopaking, Nun Zairina, Widjoseno Gardjito, Inge C Gyssens

**Affiliations:** 1 Department of Internal Medicine, Dr. Soetomo Hospital - School of Medicine, Airlangga University, Surabaya, Indonesia; 2 Department of Infectious Diseases, Leiden University Medical Centre, Leiden, The Netherlands; 3 Department of Pharmacy, Dr. Soetomo Hospital - School of Medicine, Airlangga University, Surabaya, Indonesia; 4 Department of Urology, Dr. Soetomo Hospital - School of Medicine, Airlangga University, Surabaya, Indonesia, deceased; 5 Department of Medical Microbiology & Infectious Diseases, Canisius-Wilhelmina Hospital, Nijmegen, The Netherlands; 6 Department of Medicine, Nijmegen Institute for Infection, Inflammation, and Immunity (N4i), Radboud University Nijmegen Medical Centre, Nijmegen, The Netherlands

## Abstract

**Background:**

Antimicrobial resistance is an increasing problem in developing countries and antibiotic use is widespread. Our previous surveys in Java, Indonesia, revealed that most antibiotic use was probably unnecessary or ineffective. The aim of this study was to explore a potential connection between resistance and substandard antibiotics sold in the area.

**Methods:**

A cross-sectional field study using the *simulated client *method was conducted in Surabaya. Five first-line antibiotics were requested with or without prescription (OTC). A certified laboratory analysed the drug content using validated methods. Possible determinants of substandard quality were explored.

**Results:**

In total, 104 samples from 75 pharmacies, ten drug stores and 39 roadside stalls (kiosks) were obtained. Pharmacy employees filled all OTC requests. Three quarters of kiosks sold antibiotics. Antibiotics were dispensed as single blister strips or repackaged (16%) without label. Ninety five percent of samples carried the label of 14 Indonesian manufacturers. The pharmaceutical quality did not meet BP standards for 18% of samples. Deviations (less active ingredient) were small. There was no association between low content and type of outlet, sold with or without prescription, registration type, price or packaging. Median retail prices of products carrying the same label varied up to 20 fold.

**Conclusions:**

Antibiotics were available OTC in all visited pharmacies and sold in the streets of an Indonesian city. Most samples contained an active ingredient. We urge to increase enforcement of existing regulations, including legislation that categorizes antibiotics as prescription-only drugs for all types of medicine outlets, to limit further selection of antimicrobial resistance.

## Background

Antimicrobial resistance is an increasing global crisis in developing countries. Antibiotic use, particularly over the counter, is widespread. Very little data substantiate this problem. We surveyed antibiotic usage and resistance in two areas on Java, Indonesia [1] in individuals who visited public healthcare facilities. Those who could cite the name and dosing regimens of antibiotics had consumed short courses (median three days) of amoxicillin or ampicillin, chloramphenicol, ciprofloxacin, cotrimoxazole, and tetracycline. The antibiotics were prescribed by doctors in public hospitals (12%), healthcare centers (29%), private practice (36%), and by nurses and midwives (6%). Similar antibiotics were self-medicated by 17% of the individuals. Antibiotics were purchased from community pharmacies, drug stores, traditional Chinese medicine prescribers (shinshes) or their shops and kiosks (roadside stalls, usually on wheels) in the areas surrounding the healthcare facilities. Low dosage regimens were reported [2]. Among 3275 individuals (community 2494, hospital 781), 54% carried resistant *Escherichia coli*. Recent antibiotic use was the most important determinant of resistance in both populations [community: odds ratio (OR) 1.8, 95% confidence interval (95% CI) 1.5-2.3; hospital: OR 2.5, 95% CI 1.6-3.9] [3]. We hypothesized that the low-dose regimens of a limited number of classes could have contributed to the development of resistance. In addition, if antibiotics on the market in this area have a low content, this would further expose bacteria to low concentrations and select for resistance in this population. If, on the other hand, some products are counterfeit and contain no antibiotic at all, the conclusions of our studies of the association between use and resistance in the area would be invalid.

Substandard and counterfeit anti-infective drugs can cause therapy failure because of low dosage or absence of active drug, adverse effects through excessive dose or the presence of incorrect or toxic ingredients, and emergence of antimicrobial resistance through subtherapeutic amounts of the antimicrobial drugs [4]. A substandard drug is a drug that fails to meet the specifications upon laboratory testing in accordance with the specifications it claims to comply with. Drugs can be substandard among others because they are produced with low quality or chemically instable ingredients or stored in inadequate conditions. According to the WHO definition, a counterfeit drug is one that is deliberately and fraudulently mislabeled with respect to identity, source, or both [5,6]. Counterfeit products can contain the correct amount, too little or too much of the active ingredient. Packaging can provide clues about counterfeiting [7]. The problem of substandard and counterfeit drugs is particularly prevalent in low-income and developing countries. Insufficient regulation and manufacturing control are major causes [5]. In the last decade, substandard anti-malarial, antibacterial, and antiviral drugs have been reported in South East Asia [8-10]. There are plenty of reviews and reflection papers [11-13], but recent reliable data on the prevalence of substandard drugs are lacking [14]. Recently, experts called for more political will and less secrecy and for reporting of the manufacturer's name as stated on the packaging [4].

There are very few publications on the quality of anti-infective drugs on the market in Indonesia [15,16]. As is common in many low-income countries, drugs are purchased in private drug outlets. Silverman et al. and Lee et al. stated in 1990 [15] and 1991[17], respectively, that in Indonesia, fraudulent drug products may represent 20-30% of all drug products on the market. However, no methodological details were provided. The Indonesian Drug and Food Control Agency (BPOM) reported that antibiotics are amongst the most commonly counterfeited drugs. In 2003, BPOM discovered 55 counterfeit medicines being sold in the market. Among them were amoxicillin 500 mg capsules that contained only 45.84% of the active ingredient [18]. Drug legislation and regulation have improved in Indonesia. In 2003 the government updated regulations regarding good manufacturing practice for the many local private and state companies that produce medicines, mostly generics [19]. The law categorizes antibiotics as prescription-only drugs to be retailed in pharmacies only [20]. Pharmacies and drug stores need a government license. Pharmacies are allowed to sell all categories of drugs. Drug stores have a limited license and are not allowed to dispense prescription-only drugs and narcotics. Kiosks can only sell "free drugs", e.g. vitamins and paracetamol. The strategic report *Indonesia Pharmaceutical and Healthcare Report Q3 2006 *and the Matrix of Drug Quality Report (update 2008) mentioned that fake products accounted for up to 20-25% of total drug sales [18,21].

One method to study medicine dispensing is to use *simulated clients*, a method widely practiced by both consumer organisations and researchers [22]. We choose this method to obtain independent and unbiased information on availability and quality of drugs in our setting.

In this paper we address the following two questions: To what extent are first-line antibiotics available with or without prescription in Indonesia, and secondly: what is the pharmaceutical quality of the active ingredients obtained in these ways?

## Methods

### Study design

A cross sectional survey of potential retailers of antibiotics was conducted within the framework of the AMRIN Study [1-3]. The protocol of the AMRIN study was approved by the Dr. Soetomo Hospital ethics committee (ethical clearance No.5/Panke.KKE/2001 (Surabaya). The study area was the city of Surabaya, East Java, in the streets surrounding the government hospital and two public health centres that had participated in our previous [2] antibiotic survey. In this survey, 417 users of antibiotics cited community pharmacies drug stores, traditional Chinese medicine prescribers (shinshes) or their shops and kiosks (roadside stalls, usually on wheels) as providers of the antibiotics that they used. The typical profile of the OTC user was adult, male and without health care insurance. Therefore, four volunteers (three men and one woman) were trained to act as clients purchasing antibiotics in these types of outlets. They requested the first-line antibiotics with (pharmacy only) or without prescription (all outlets) by the generic name, as is the custom in Indonesia. Prescriptions for three-day courses were made by a physician for the purpose of the study and presented by the clients in pharmacies only. Unfilled prescriptions were destroyed. When questioned, the clients were instructed to cite standard symptoms of infection, e.g. fever, cough, pain while passing urine, etc.

### Sample collection

**Figure 1:**
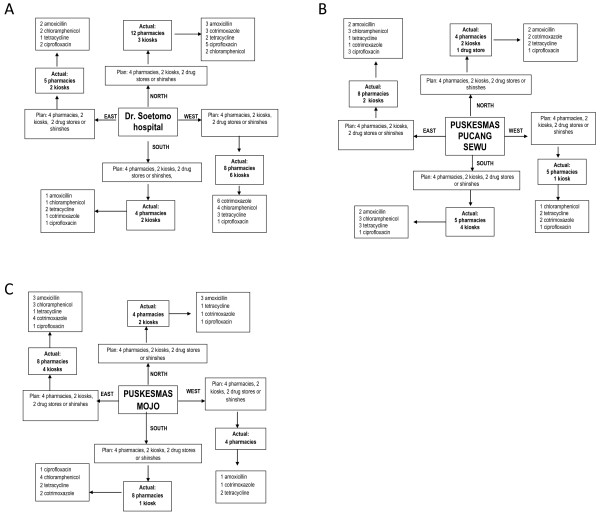
**Study plan and actual sample collection of antibiotics from medicine outlets in areas surrounding public healthcare facilities by *simulated clients *in the city of Surabaya**. 1a Itineraries in the area surrounding the public hospital Dr. Soetomo. 1b Itineraries in the area surrounding the public healthcare centre Puskesmas Pucang Sewu. 1c Itineraries in the area surrounding the public healthcare centre Puskesmas Mojo.

No reliable list of registered pharmacies, drugstores or kiosks of the city of Surabaya was available to us at the time of the survey. A field sampling schedule was designed (Figures 1a, b, c). Starting from the government hospital and two public health centres that were included in the previous survey [2], the four 'clients' had to follow a distinct direction (north, west, south, and east) and visit each consecutive retailer on the way. Each retailer was visited once. A first sample size of 100 purchases was deemed appropriate as a pilot, consisting of 20 samples of the most used five first-line antibiotics (amoxicillin, chloramphenicol, tetracycline, cotrimoxazole, or ciprofloxacin) each, with or without a prescription. If large differences in content were found, the plan would be to increase the sample size. The following information was recorded: date of purchase; name and address of retailer; type of retailer; obtained with or without prescription; storage conditions of the antibiotic; type of packaging; delivered with or without information leaflet (package insert) or oral instruction, and price. After the survey, a list of registered pharmacies per district obtained from a local pharmaceutical company was used to estimate the coverage of our survey.

### Analysis of samples

Samples were digitally photographed (camera Nikon E7900, 3072 × 2304), including package and information leaflet, when available. All samples were used for analysis. Assays of tablets and capsules were performed by Farmalyse BV (Zaandam, the Netherlands), an ISO 9001: 2000 certified laboratory registered in the European Union as a pharmaceutical control laboratory for chemical physical analyses by license nr 101037. High performance liquid chromatography (HPLC) (Thermo Electro Scientific, Breda, The Netherlands) was used. The tests were performed in duplicate and executed in conformity with GMP-GCLP guidelines (Annex 14 + 15 GMP guidelines). A separate quality assurance unit reviewed the final report. For content of active ingredient assays of cotrimoxazole, tetracycline, and chloramphenicol the British Pharmacopoeia BP2005 monographs and standards were used [23], for ciprofloxacin and amoxicillin the United States Pharmacopeia USP 29 [24].

### Statistical analysis

SPSS for Windows version 13 was used for all analyses. Fisher's exact test was used to analyze the association between substandard quality (using BP limits) [23] and retailer, storage conditions, packaging, stated manufacturer, whether the product was registered as generic or branded, sold with or without prescription and price (> 20% above price set by the government). Prices in pharmacies and kiosks and of generic and branded products were compared by the Wilcoxon signed-rank test.

## Results

### Medicine outlets and availability of antibiotics

Between 7 and 15 April 2006, the four 'clients' requested an antibiotic from the potential medicine retailers in the city of Surabaya along the 3 × 4 routes of the study plan. Figure 1a shows the yield on first route, departing from the hospital. Details on the second and third route are shown in Figure 1b and 1c. Only one of the first ten drug stores and none of the four shinshes/traditional Chinese medicine shops encountered sold antibiotics. From that moment, samples primarily planned to be purchased in drug stores or from shinshes were requested without prescription in kiosks or pharmacies. According to the list of the licensed community pharmacies provided by the pharmaceutical company, the study sample covered 3-57% of 12 of 31 sub-districts in Surabaya. Overall, 12% of the 655 licensed pharmacies in Surabaya were visited. All but one was a private pharmacy and all had air conditioning. Total numbers of drug stores, shinshes and kiosks in Surabaya were not available, making it impossible to calculate the proportion sampled for these retailers. In pharmacies, the requested amount of product was dispensed from boxes (blisters) or bottles of 500 to 1000 units. All antibiotics and quantities were available, except for one pharmacy that dispensed only 17 capsules of chloramphenicol instead of 20. In kiosks, the antibiotics were exposed to sunlight, average high Indonesian temperatures of 32°C and high humidity. In six kiosks the requested number of units was not available (four to seven instead of ten units). Ten kiosks out of 39 did not sell antibiotics.

### Antibiotic samples and dispensing

**Figure 2:**
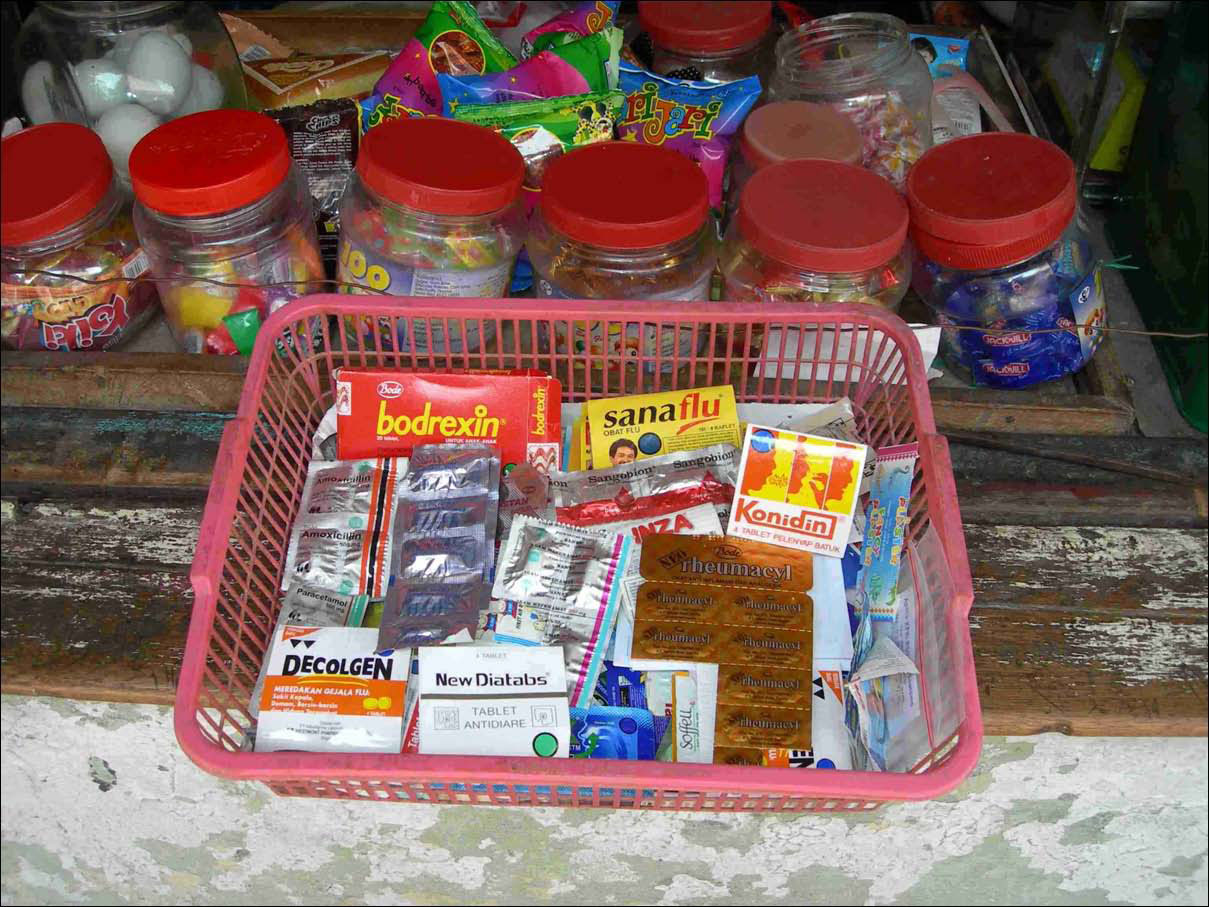
**Antibiotic sample in a kiosk (roadside stall) in a street of Surabaya**. Photograph by Usman Hadi.

In Table 1 the characteristics of the purchased antibiotics are shown. All samples with prescription were acquired from pharmacies. In case of a prescription, pharmacy employees copied dose regimens from the prescriptions onto a slip of paper and attached it to the package. No information leaflets or oral instruction were given. In case of purchase without prescription, the clients were never questioned or referred to a physician. Both in pharmacies and kiosks, products with similar appearance and packaging (according to visual inspection and comparison of the high resolution digital photographs) were dispensed. Figure 2 shows a blister strip of amoxicillin sold in a kiosk. Most samples were generic products. Twenty-two percent of chloramphenicol, 15% of cotrimoxazole, and 60% of tetracycline samples were branded generics. Capsules were repackaged in small plastic bags, 16% of which did not have a label with the drug name or unit dose. For 95% of the samples, the tablets and capsules carried the labels of 14 Indonesian manufacturers (Table 2).

**Table 1:** Characteristics of antibiotic products purchased by simulated clients at medicine outlets in Surabaya, Indonesia

Antibiotic samples	Amoxicillin 500 mg (10 tablets) N = 20	Chloramphenicol 250 mg (20 caps.^c^) N = 23	Ciprofloxacin 500 mg (10 tablets) N = 19	Co- trimoxazole 480 mg (10 tablets) N= 20	Tetracycline 250 mg (10 caps.) N = 19	Tetracycline 500 mg (10 caps.) N = 3	Total N = 104
Medicine outlet							

pharmacy/kiosk/drug store	9/11/0	19/4/0	17/2/0	20/0/0	8/10/1	2/1/0	75/28/1

Prescription							

yes/no	5/15	5/18	5/14	6/14	4/15	1/2	25/79

Packaging							

blister/plastic bag	20/0	14/9	19/0	20/0	12/7	2/1	87/17

Registration							

generic/branded generic	20/0	18/5	19/0	17/3	7/12	2/1	83/21

Price^a^/sample							

range	290-5000	150-1000	400-8000	145-870	138-1000	300-750	138-8000
median (IQR)^b^	500 (313-700)	325 (250-500)	850 (500-850)	223 (200-405)	800 (150-900)	400(300-750)	500 (250-800)

^a^In rupiah, ^b^Interquartile range, ^c^one pharmacy dispensed 17 capsules

**Table 2:** Stated manufacturer (city of residence) on blisters and/or logos for 104 samples and substandard content assay result

	Substandard assay test results/total					Total
	
Stated to be manufactured by (city)	Amoxicillin	Chloramphenicol	Ciprofloxacin	Cotrimoxazole	Tetracycline	
Aditama Raya Farmindo (Surabaya)	-	-	-	1/1	-	1/1

Bernofarm (Sidoarjo)	-	-	0/3	-	-	0/3

Darya Varia Laboratoria (Bogor)	-	-	-	-	3/11	3/11

Dexa Medica (Palembang)	-	-	0/10	-	-	0/10

Dumex (Jakarta^a^)	-	-	-	-	1/1	1/1

ERBA/Kalbe Farma (Bekasi^a^)	-	0/2	-	-	-	0/2

First Meditama (Sidoarjo)	-	-	-	0/1	-	0/1

Indofarma (Bekasi)	1/4	0/10	0/2	8/9	0/3	10/28

Kimia Farma (Jakarta)	0/1	0/2	0/1	0/3	0/2	0/9

Landson PT Pertiwi Agung (Jakarta^a^)	-	0/1	-	-	-	0/1

Phapros (Semarang)	2/14	-	-	0/1	0/2	2/17

Saka Farma (Jakarta^a^)	-	0/2	-	0/1	-	0/3

Sanbe Farma (Bandung)	-	0/4	0/3	0/3	-	0/10

Yarindo (Serang)	1/1	-	-	1/1	-	2/2

Unknown	-	0/2^b^	-	-	1/3^c^	1/5

Total	4/20	0/23	0/19	10/20	5/22	19/104

^a^Head office address, ^b^In small plastic bags and no text or logo on white/green capsules, ^c^In plastic bags and no text or logo on red and black caps.; red and black caps. "BMF"; yellow and blue capsules "TETRA 500"

### Content assay and analysis

Antibiotic samples were collected and stored in an air-conditioned room until transportation for analysis to the Netherlands in the third week of April 2006. According to the BP2005 criteria, one fifth of amoxicillin tablets and 5 of 22 tetracycline capsules samples contained slightly less active substance than required (Table 3). Fifty percent of cotrimoxazole tablets had a trimethoprim content which was not meeting BP standards; deviations were up to 20% of the required amount. None of the samples had excess active ingredient. No association between low content and type of outlet (p = 0.58), sold with or without prescription (p = 0.78), packaging (p = 0.8) registration type (generic versus branded) (p = 0.54) or price (p = 0.33) was observed. Substandard antibiotics carried the labels of six manufacturers (Table 2). On six out of ten substandard products a legible expiry date was printed on the blister package, in all cases beyond January 2008.

**Table 3:** Antibiotic content of 104 oral samples purchased in medicine outlets in Surabaya, Indonesia

Antibiotic	Required active substance (%, min, max)	Number of samples		Content of active substance mg (% of stated dosage)/tablet or capsule		Content too low^a^
		
		Pharmacy	Kiosk/Drug store	Min	Max	N (%)
Amoxicillin 500 mg tablet	92.5 - 110	9	11	457 (91.4)	481 (96.2)	4 (25)^b^

Chloramphenicol 250 mg capsule	95 - 105	19	4	239 (95.6)	260 (104)	0

Ciprofloxacin 500 mg tablet	95 - 105	17	2	481 (96.2)	506 (101.2)	0

Cotrimoxazole 400/80 mg tablet sulfamethoxazole trimethoprim	92.5 - 107.5 92.5 - 107.5	20	0	391 (97.8) 58 (72.5)	411 (102.8) 81 (101.3)	0 10 (50)^c^

Tetracycline 250 mg capsule	95 - 105	8	10	226 (90.4)	259 (103.6)	5 (23)^d^

Tetracycline 500 mg capsule	95 - 105	2	1	503 (100.6)	514 (102.8)	0

^a^Compared with pharmacopeial limits [23]

^b^Two were obtained from a pharmacy; ^c^All were obtained from a pharmacy; ^d^One was obtained from a pharmacy

### Retail prices

In Table 4 a retail price comparison is given, including the price set by the government [25]. Generic products were less costly than branded generic products; for chloramphenicol, cotrimoxazole, and tetracycline the differences were statistically significant. In kiosks, retail prices were significantly higher than in pharmacies. In pharmacies, there was no association between retail price and lack of a prescription (data not shown).

**Table 4:** Retail price comparison of the antibiotics

Antibiotics	Recom- mended^a^ retail price^b^	Actual retail price^b ^per capsule or tablet median (IQR)					
		
		In pharmacies	In kiosks	p-value	Generic	Branded generic	p-value
Amoxicillin 500 mg tablet	403	300 (300-400)	700 (500-1000)	0.00	500 (313-700)	Not available	-

Chloramphenicol 250 mg capsule	237	258 (230-470)	875 (563-1000)	0.00	254 (224-500)	500 (460-875)	0.00

Ciprofloxacin 500 mg tablet	950	850 (500-1000)	5000 (2000-8000)	0.00	850 (500-1000)	Not available	-

Cotrimoxazole 480 mg tablet	205	223 (200-405)	Not available	-	220 (200-260)	850 (200-870)	0.00

Tetracycline 250 mg capsule	125	150 (150-200)	900 (800-1000)	0.00	150 (150-200)	850 (800-1000)	0.00

Tetracycline 500 mg capsule	266	350 (300-400)	750 (750-750)	0.17	350 (300-400)	750 (750-750)	0.17

^a ^Maximum retail price set by the Minister of Health in March 2006 [25]

IQR= interquartile range

^b^ In rupiah

## Discussion

This field study showed that, despite existing regulations, five first-line antibiotics could easily be obtained without a prescription from medicine retailers in one of the major cities in Indonesia. These antibiotics were available in all community pharmacies visited. All pharmacies and three quarters of roadside stalls sold antibiotics OTC, suggesting that the problem is widespread. Apart from cotrimoxazole, most samples showed the expected amounts of active ingredient.

The availability of antibiotics in other medicine outlets, that are not licensed for prescription-only drugs, differed. Drug stores hardly retailed any antibiotics. This was confirmed by a repeat visit to a few drug stores in 2007 (data not given). Former actions by the national Drug and Food Control Agency towards drug stores might have been effective. Distribution and sales of illegally imported drugs is punishable by up to five years of imprisonment and a fine of two billion rupiah (around 140,000 EUR).

One of the reasons for OTC retailing in kiosks could be that, having no fixed address, kiosk owners escape control. Although there is no medical need for kiosks to retail antibiotics for the purpose of availability - for emergencies 24-hour coverage is given by hospital pharmacies-, it is clear that kiosks fill a commercial demand. Kiosks are convenient because they are numerous in urban areas, easily accessible and pose no privacy problems for the nightly client with e.g. a sexually transmitted disease. Tetracyclines are the most frequently obtained antibiotics OTC [2]. Consumers may not be aware that resistance against tetracyclines in *Neisseria gonorrhoeae *has approached 100% in urban areas since the nineties [26]. Kiosk owners used to buy their drugs from wholesalers or drug stores. It now seems that, because of recently tightened control of wholesalers and drug stores, kiosk owners obtain small quantities of antibiotics from nearby pharmacies. Strict enforcement of the law for community pharmacies without controlling the kiosks might make owners turn to an alternative illegal supply chain of anti-infectives again. Illegal import increases the risk of counterfeit products in other South East Asian countries [10,27,28]. This study confirms the data of our previous survey [2] and statements in the press [29] that Chinese traditional healers do not retail antibiotics.

The lack of appropriate labeling and counseling in pharmacies can be partly explained by the insufficient level of training of pharmacy employees, which does not include information on drugs. Other reasons for insufficient labeling and information could be the practice of repackaging large (cheaper) packaged units, without providing (copies of) information leaflets.

The fact that eight out of nine cotrimoxazole samples stated to be manufactured by one local company were substandard, out of a total of ten substandard samples, points to a production problem. Also, because no product carried a date beyond expiration, this was considered as an unlikely cause of the low content. Although exposure to sunlight, average high temperatures of 32°C in Indonesia and high humidity could have led to degradation of drugs in kiosks, we found no association of substandard content of the products with the type of medicine outlet. We cannot prove that the antibiotic samples were produced by the companies printed on the labels. However, we have no indication that any of the samples were counterfeit drug products. Products purchased in licensed pharmacies and kiosks carried the same label and all samples contained active ingredients. In developing countries that struggle with counterfeit drug problems, some samples contained no active ingredient at all, too high or very low amounts [8,10,30]. Using similar methods, researchers from Burma found the antibiotic content of 21 products to be 13 to 48% lower than expected [10]. In Laos People's Democratic Republic, ampicillin and tetracycline contained 3 to 32% and 8 to 14% less active ingredient than expected [9]. In the present study, apart from cotrimoxazole (maximally 20% less active ingredient), deviations from the BP2005 limits were small. As little variation in availability and active ingredient of the antibiotics was found in the first 104 samples, this sample size was considered sufficient for the total survey. Another recent study in Indonesia showed a slightly lower content of rifampicin in products from one out of three manufacturers [16]. Therefore, we cannot concur with former published statements [15,17,18,21] on fraudulent drugs in Indonesia, although we cannot exclude that the situation has improved recently or that the situation in Surabaya is different from other areas in Indonesia. The regulations seem to at least effectively guarantee the presence of genuine antibiotics on the market.

Widespread unjustified use of the first line antibiotics [2,31] is expected to be the major avoidable contributor to increasing resistance. The hypotheses leading to this study were whether active ingredient in antibiotics purchased in the area was too low or absent. Low active ingredient (which might contribute to the development of the resistance of bacteria) was only found for cotrimoxazole tablets. Interestingly, the strongest association of antibiotic use with resistance found in the community population in our previous survey was prior use of cotrimoxazole. It was associated with carrying *E. coli *resistant to any of the tested antibiotics (OR 5.5, 95% CI 2.1-14.8) and single trimethoprim/sulfamethoxazole resistance (OR 7.5, 95% CI 2.0-28.0) [3]. However, we should keep in mind that the samples of the present study were obtained only in one out of the two areas of the previous survey, in a different time period and that we have no information on brand/batches that were consumed by the individuals who carried the resistant *E. coli*.

Prices of antibiotics with similar labels differed widely, often exceeding the recommended [19] retail price, except for generic amoxicillin and ciprofloxacin (Table 4). Generic products were less costly than branded products, and prices were lower in pharmacies than in kiosks.

This is the first published report in the international literature that provides objective information on OTC sales and the active ingredient of frequently used oral antibiotics in Indonesia. The strengths of the study are the large sample size of essential antibiotics from one stated country of origin (Indonesia) and the analysis by a laboratory using HPLC, which is industry standard for testing for the amount of active ingredient. Because of the *simulated client *method, commonly used in similar studies to procure drug samples for testing [8,28,30], we were able to document real life practices. A more official approach of drug quality assurance assessment including drug pricing was recently developed by WHO [32]. However, this method, which also requests consent of the responsible retailers [33], would not have exposed the OTC sales problem.

The present study has some limitations. We were not able to select pharmacies using random sampling [8,10,28,30] from an official list of registered pharmacies in Surabaya; however, our field survey approach should exclude major bias. We assume that, because samples and packages of the same antibiotics sold in licensed pharmacies and kiosks looked genuine and very similar by visual inspection and by comparison of the digital photographs, they were not counterfeit. We did not present the samples to the stated manufacturing companies or to the regulatory authority, which may have more experience and/or technology to make this determination. However, the chairman of the Indonesian Pharmacists Association (ISFI) recently said " that it was theoretically impossible for counterfeit drugs to be taken up by hospitals or licensed pharmacies because they normally obtained their medicines through legal distributors"[34]. We were not systematically informed about the expiry dates of the samples because this information was missing for the repackaged products or absent from the partial blister strips. However, no legible dates were beyond expiration. We selected the antibiotics based on their substantial usage in the area [2], not on high cost. Therefore, we cannot generalize our findings to all types of antibiotics, since expensive antibiotics might be a more interesting target for counterfeiters. However, large volumes of inexpensive fakes would still provide a worthwhile profit for counterfeiters [4]. We think that our findings are relevant for the urban areas as very similar antibiotic consumption patterns were found in the other city of our survey on Java [2]. In addition, the stated manufacturers were Indonesian companies from 9 different cities of Java or Sumatra which are the main islands. Whether the results are true for rural areas or small islands of Indonesia remains to be seen. We analyzed two oral dosage forms only and did not try to study liquid preparations. Our analysis in terms of quality was limited to the content, since we did not perform analyses of impurities or excipients which determine the biological equivalence [24]. We did not perform dissolution tests [35] which would assess the in vitro availability of the drug. We did not perform follow up experiments to determine whether the samples from the kiosks, being exposed to heat and humidity, would be degraded before the expiry date.

Finally, in concert with other published data from the AMRIN study [1-3,31] that provided information on behavioral, cultural and socioeconomic determinants driving antimicrobial use and resistance on the level of the prescriber and user, this study helps to fill the knowledge gap of determinants on the level of government health policies and the health care system in which they are implemented in Indonesia. The World Health Organization Global Strategy for the Containment of Antimicrobial Resistance report [36] states that "National commitment to understand and address the problem and the designation of authority and responsibility are prerequisites for interventions. Effective action requires the introduction and enforcement of appropriate regulations."

## Conclusions

Despite these limitations, we conclude that OTC retailing of five first-line oral antibiotics was widespread in pharmacies and in kiosks of a major city on Java, Indonesia, often at very high prices. The active ingredient was lowest for trimethoprim in cotrimoxazole tablets, which most likely was related to manufacturing problems. We recommend increased enforcement of existing regulations for all types of medicine outlets by the national Drug and Food Control Agency, better training of pharmacy personnel, control of antibiotic production on a national level by local industries and pricing according to WHO expert reports [32,33].

## Competing interests

The authors declare that they have no competing interests.

Main sponsor: The Royal Netherlands Academy of Arts and Sciences (KNAW), within the framework of the Scientific Programme Indonesia-Netherlands (SPIN).

Additional sponsors: Leiden University Medical Centre (LUMC), Gilead/UCB Pharma, The Netherlands, Merck Sharp & Dohme bv, The Netherlands, and Bristol Myers Squibb bv, The Netherlands.

The sponsors of the study had no role in study design, data collection, data analysis, data interpretation, or writing of the manuscript. The additional sponsors provided small grants for the laboratory analysis.

## Authors' contributions

IG and PJB conceived the study. UH designed the study with contributions from all authors. UH and PJB did the statistical analyses. All authors contributed to data interpretation and preparation of the manuscript. The corresponding author had full access to all data and final responsibility to submit for publication.

## Pre-publication history

The pre-publication history for this paper can be accessed here:

http://www.biomedcentral.com/1471-2334/10/203/prepub

## References

[B1] Lestari ES Severin JA Filius PMG Kuntaman K Duerink DO Hadi U Wahjono H Verbrugh HA Antimicrobial resistance among commensal isolates of *Escherichia coli *and *Staphylococcus aureus *in the Indonesian population inside and outside hospitals Eur J Clin Microbiol & Infect Dis 2008 27 45 51 10.1007/s10096-007-0396-z 17934766

[B2] Hadi U Duerink DO Lestari ES Nagelkerke NJ Werter S Keuter M Suwandojo E Rahardjo E Van den Broek PJ Gyssens IC Survey of antibiotic use of individuals visiting public healthcare facilities in Indonesia Int J Infect Dis 2008 doi: 10.1016/j.ijid.2008.01.002 18396084 10.1016/j.ijid.2008.01.002

[B3] Duerink DO Lestari ES Hadi U Nagelkerke NJ Severin J Verbrugh HA Keuter M Gyssens IC van den Broek PJ Determinants of carriage of resistant *Escherichia coli *in the Indonesian population inside and outside hospitals J Antimicrob Chemother 2007 60 377 384 10.1093/jac/dkm197 17595290

[B4] Newton PN Green MD Fernandez FM Day NP White NJ Counterfeit ant-infective drugs Lancet Infect Dis 2006 6 602 613 10.1016/S1473-3099(06)70581-3 16931411

[B5] World Health Organization (WHO) What encourages counterfeiting of drugs? http://www.who.int/medicines/services/counterfeit/faqs/15/en/index.html

[B6] WHO WHO Counterfeit Drugs. Guidelines for the Development of Measures to Combat Counterfeit Drugs 1999 Geneva: WHO 1 60

[B7] Hall KA Newton PN Green MD Vandenabeele P Pizzanelli D Mayxay M Dondorp A Fernandez FM Characterization of counterfeit artesunate antimalarial tablets from Southeast Asia Am J Trop Med Hyg 2006 75 804 811 17123969

[B8] Shakoor O Taylor RB Behrens RH Assessment of the incidence of substandard drugs in developing countries Trop Med Int Health 1997 2 839 845 10.1046/j.1365-3156.1997.d01-403.x 9315042

[B9] Syhakhang L Stalsby Lundborg C Lindgren B Tomson G The quality of drugs in private pharmacies in Lao PDR: a repeat study in 1997 and 1999 Pharm World Sci 2004 26 333 338 10.1007/s11096-004-0558-3 15683103

[B10] Prazuck T Falconi I Morineau G Bricard-Pacaud V Lecomte A Ballereau F Quality control of antibiotics before the implementation of an STD program in Northern Myanmar Sexually Transmitted Diseases 2002 29 624 627 10.1097/00007435-200211000-00002 12438896

[B11] Jack A Counterfeit medicines. Bitter pills BMJ 2007 335 7630 1120 1121 10.1136/bmj.39412.431655.AD 18048535 PMC2099530

[B12] Kelesidis T Kelesidis I Rafailidis PI Falagas ME Counterfeit or substandard antimicrobial drugs: a review of the scientific evidence J Antimicrob Chemother 2007 60 2 214 236 10.1093/jac/dkm109 17550892

[B13] Caudron JM Ford N Henkens M Mace C Kiddle-Monroe R Pinel J Substandard medicines in resource-poor settings: a problem that can no longer be ignored Trop Med Int Health 2008 13 8 1062 1072 10.1111/j.1365-3156.2008.02106.x 18631318

[B14] Senior K Global health-care implications of substandard medicines Lancet Infect Dis 2008 8 11 666 10.1016/S1473-3099(08)70241-X 18992395

[B15] Silverman H Lydecker M Lee PR The drug swindlers Intern J Health Serv 1990 20 561 572 10.2190/P32D-0141-M86B-F7AT 2265874

[B16] Van Crevel R Nelwan RH Borst F Sahiratmadja E Cox J van der Meij W de Graaff M Alisjahbana B De Lange WC Burger D Bioavailability of rifampicin in Indonesian subjects: a comparison of different local drug manufacturers Int J Tuberc Lung Dis 2004 8 500 503 15141745

[B17] Lee PR Lurie P Silverman MM Lydecker M Drug promotion and labeling in developing countries: an update J Clin Epidemiol 1991 44 49S 55S 10.1016/0895-4356(91)90113-N 2045842

[B18] McGinnis M Primo-Carpenter J Matrix of drug quality reports affecting USAID-assisted countries Drug Quality Information Program 2008 The United States Pharmacopeial Convention. Rockville, MD

[B19] Anonymous Decree of the Head of National Agency of Drug and Food Control Republic of Indonesia on Criteria and Procedure of Drug Registration No. HK.00.05.3.1950. Jakarta 2003

[B20] Anonymous Undang-Undang Obat Keras Staatsblad no 419 1949

[B21] International Business Monitor Indonesia Pharmaceuticals and Healthcare Report Q3 2006 London, UK 2007 1 65

[B22] Norris PT Purchasing restricted medicines in New Zealand pharmacies: results from a "mystery shopper" study Pharm World Sci 2002 24 4 149 153 10.1023/A:1019506120713 12227248

[B23] British Pharmacopoeia Commission British Pharmacopoeia 2005 2005 The Stationery Office

[B24] United States Pharmacopeial Convention The United States Pharmacopeia USP 29 2006

[B25] The Minister of Health Decree on the retail prices of generic drugs No. 156/MENKES/SKIII/2006. Jakarta 2006

[B26] Ieven M Van Looveren M Sudigdoadi S Rosana Y Goossens W Lammens C Meheus A Goossens H Antimicrobial susceptibilities of *Neisseria gonorrhoeae *strains isolated in Java, indonesia Sexually Transmitted Diseases 2003 30 25 30 10.1097/00007435-200301000-00006 12514438

[B27] Dondorp AM Newton PN Mayxay M Van Damme W Smithuis FM Yeung S Petit A Lynam AAJ Johnson A Hien TT Fake antimalarials in Southeast Asia are a major impediment to malaria control: multinational cross-sectional survey on the prevalence of fake antimalarials Trop Med Int Health 2004 9 1241 1246 10.1111/j.1365-3156.2004.01342.x 15598255

[B28] Lon CT Tsuyuoka R Phanouvong S Nivanna N Socheat D Sokhan C Blum N Christophel EM Smine A Counterfeit and substandard antimalarial drugs in Cambodia Trans Royal Soc Trop Med Hyg 2006 100 1019 1024 10.1016/j.trstmh.2006.01.003 16765399

[B29] AFO Chinese medicine stores retain their lure in Indonesia Kabar-indonesia 2006 Jakarta Joyo Press Service

[B30] Taylor RB Shakoor O Behrens RH Everard M Low AS Wangboonskul J Reid RG Kolawole JA Pharmacopeial quality of drugs supplied by Nigerian pharmacies Lancet 2001 357 1933 1936 10.1016/S0140-6736(00)05065-0 11425415

[B31] Hadi U Duerink DO Lestari ES Nagelkerke NJ Keuter M Huis in't Veld D Suwandojo E Rahardjo E van den Broek PJ Gyssens IC Audit of antibiotic prescribing in two governmental teaching hospitals in Indonesia Clin Microb Infect 2008 14 698 707 10.1111/j.1469-0691.2008.02014.x 18558943

[B32] WHO Specifications for Pharmaceutical preparations WHO Technical Report Series 948 2008 Geneva: WHO 18557474

[B33] World Health Organization Measuring medicine prices, availability, affordability and price components Report No.: WHO/PSL/PAR/2008.3

[B34] Sagita D Rachman A Raids Uncover Counterfeit Drugs JakartaGlobe J17 March 2009, akarta

[B35] Risha PG Shewiyo D Msami A Masuki G Vergote G Vervaet C Remon JP *In vitro *evaluation of the quality of essential drugs on the Tanzanian market Trop Med Int Health 2002 8 701 707 10.1046/j.1365-3156.2002.00937.x 12167097

[B36] World Health Organization WHO Global strategy to contain antimicrobial resistance 2001 Geneva, Switzerland: WHO

